# Low-Dimensional Motor Control Representations in Throwing Motions

**DOI:** 10.1155/2017/3050917

**Published:** 2017-12-31

**Authors:** Ana Lucia Cruz Ruiz, Charles Pontonnier, Georges Dumont

**Affiliations:** ^1^INRIA/IRISA/M2S MimeTIC, Rennes, France; ^2^Ecole Normale Supérieure de Rennes, Univ Rennes, Rennes, France; ^3^Ecoles de Saint-Cyr Coëtquidan, Guer, France

## Abstract

In this study, we identified a low-dimensional representation of control mechanisms in throwing motions from a variety of subjects and target distances. The control representation was identified at the kinematic level in task and joint spaces, respectively, and at the muscle activation level using the theory of muscle synergies. Representative features of throwing motions in all of these spaces were chosen to be investigated. Features were extracted using factorization and clustering techniques from the muscle data of unexperienced subjects (with different morphologies and physical conditions) during a series of throwing tasks. Two synergy extraction methods were tested to assess their consistency. For the task features, the degrees of freedom (DoF), and the muscles under study, the results can be summarized as (1) a control representation across subjects consisting of only two synergies at the activation level and of representative features in the task and joint spaces, (2) a reduction of control redundancy (since the number of synergies are less than the number of actions to be controlled), (3) links between the synergies triggering intensity and the throwing distance, and finally (4) consistency of the extraction methods. Such results are useful to better represent mechanisms hidden behind such dynamical motions and could offer a promising control representation for synthesizing motions with muscle-driven characters.

## 1. Introduction

Understanding how humans control motion is an important aspect in a variety of fields, ranging from neuroscience to robotics and animation [1]. Several theories have been proposed which aim at unveiling the efficient and powerful mechanisms behind human motion generation. In neuroscience and biomechanics, some of the objectives of identifying such mechanisms are to validate an existing motor control theory, to diagnose and treat pathologies, or to enhance athletic performance. In animation and robotics, identifying such mechanisms is the key to enhance the realism and efficiency of the motions in virtual humans and robots, since it would allow the development of more realistic motion controllers, reflecting a global control of motion [2]. More realistic motions imply a higher degree of similarity to humans, at the visual, kinematic, and dynamic level.

Our motivation lies in the domains of neuroscience and animation. In the animation field, characters with more detailed actuators (or muscles) are starting to be used for motion synthesis. The use of muscle-based characters entails several advantages such as smoother torque generation [3], more realistic responses to perturbations [4, 5], and an ease to simulate pathologies and fatigue [6, 7]. However, the use of muscles complicates the control problem by augmenting nonlinearity and redundancy, as at least two muscles are necessary to actuate each degree of freedom [8]. Furthermore, computationally expensive optimization-based solutions, which are unlikely to represent how humans control motions, are used to compute a high number of control signals.

Thus, it is necessary to define compact control schemes reducing the complexity of the control for such applications. Neuroscience provides several interesting ways to circumvent this issue, such as the theory of muscle synergies [1, 9], that tries to reduce redundancy by identifying a simple and generic control representation of given tasks. This theory is based on an interesting hypothesis of how redundancy is handled by the central nervous system (CNS): it assumes the existence of links between the ensemble of muscle control signals during the performance of a task, which reduces redundancy. Thus, through synergies, the muscles are controlled as groups and not individually. There is support for a neural organization of these synergies [10, 11] while remaining an open question [12, 13]. Even if the interpretation of the low dimensionality revealed by decomposition methods is subject to debate [14, 15], these methods allow the creation of a compact and low dimensional control representation based on experimental data for simple and complex motions. Indeed, electromyography (EMG) signal-processing strategies (principal components analysis, nonnegative matrix factorization) are able to extract compact features from EMG signals, even if these extracted synergies may be a consequence of a more complex mechanism [16, 17]. Many studies have extracted synergies during a variety of simple upper body human motions (such as pointing and reaching [18, 19]). However, to the authors' knowledge, only few studies have dealt with complex, unconstrained (or free), and dynamic motion [20, 21], and among them, overhead throwing is interesting to analyze through this theory.

An overhead throwing motion consists of launching an object forward and above the shoulder by using one arm. This is the type of motion with which humans can throw with speed and accuracy [22]. Unlike simple manipulation tasks such as reaching, lifting, pulling, and pushing, this task is more complex, requiring a higher coordination, accuracy, and skill. Thus, it is a highly redundant and nonlinear task, which involves a dynamic manipulation [23]. It is highly redundant because there exists an infinite number of solutions or movements that achieve the same target hit. It is highly nonlinear due to the fact that positions and velocities are coupled and that in order to hit the desired target, at the moment of ball release, the hand velocity, position, and the object's time of flight should satisfy the parabolic projectile equation. Finally, it is also dynamic because of the high accelerations and momentum at certain motion phases. Thus, the aim of this work is to identify a compact representation of the control mechanism behind overhead throwing motions in order to (1) validate a control representation extraction methodology and (2) produce a low-dimensional control representation which could later be exploited to synthesize throwing motions in animation. Such representations could later be extracted from other types of throwing motions (such as sidehand and underhand throws) and simpler arm motions such as pointing, to feed a controller library that may produce a variety of motions from these compact representations [24].

The generic representation should contain a reduced set of control variables (less than the number of joint actions in study). It should also encode important temporal and spatial control trends, invariant over a variety of morphologies. Finally, it should show some of the links between these control variables and task space goals or features, that has to be used as controller inputs in motion synthesis tools. One of our previous work [25] has shown that the control strategy could be represented as synergies during throwing motions. However, this analysis comprehended solely the activation space and a unique subject. In this paper, we propose to extend this analysis by extracting control strategies from a variety of subjects and by analyzing their relationships with kinematic goals.

For this purpose, we present an analysis to identify generic representations of control strategies starting at the task space level, joint space level, and until finally reaching the activation (or actuation) space level, where we extract the synergies or basis control functions. First, the experimental setup used to extract the motion and muscle data is presented. Next, the control variables or features at the task, joint and activation spaces are defined. This is then followed by a detailed explanation of the methods used to extract the control representation, which encompass clustering and matrix factorization techniques. Two methods were used to extract the muscle synergies and the consistency of their results is assessed. Finally, the generic control strategies representations are illustrated and explained. Results show the existence of a generic control representation at the activation level for a variety of morphologies during throwing tasks, and its relationship with task space goals and features. Our new model could be later used to control a larger variety of characters and a larger family of motions involving similar task space goals.

## 2. Materials and Methods

### 2.1. Experimental Setup

#### 2.1.1. Subjects

Ten healthy men (age 29.8 ± 5.6 years old; weight 72.4 ± 9.9 kg; height 1.77 ± 0.07 m) volunteered for the experiments. The subjects were all right handed and all except one (subject 3) had never suffered injuries in the right arm. Furthermore, none of the participants were professional athletes, and they all had different physical conditions (with a mean number of hours of sport activity per week of 3.85 ± 3.07). Each subject provided a written informed consent form before participation. The experimentation was conducted in accordance with the Declaration of Helsinki (1964). The study was approved by the ethics committee of the M2S laboratory of University of Rennes 2.

#### 2.1.2. Task

**Figure 1:**
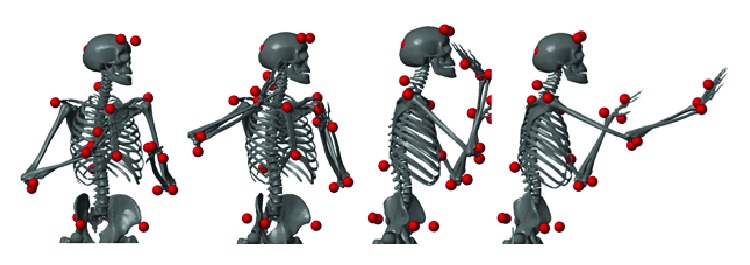
Overhead throwing motion. Example of an overhead throwing motion to a 4 m target (bone graphics issued from [27]). Representative posture of each phase of the motion is shown.

**Figure 2:**
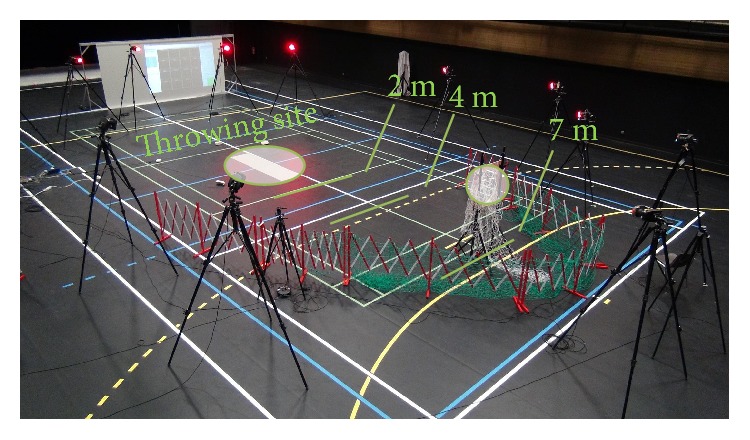
Experimental setup. The setup consisted of a throwing site and a target that could be placed at 2, 4, and 7 m from the thrower. Motion capture was done thanks to 16 cameras, and EMG measurements were done through a wireless EMG system.

A series of experiments were conducted where the task consisted of a right-hand overhead throw to a static target placed at different distances from a fixed throwing site. The target was placed at 2 m, 4 m, and 7 m along a straight line from the throwing site. The target was a hole of diameter 0.7 m placed at 1.5 m from the ground. The ball was a standard American football ball, 0.28 m long and 0.15 m large, weighing 0.4 kg. Before beginning the experiments, the subjects underwent a short training where they practiced long distance throws for 5 to 10 minutes. Once the training was finished, the experiments began. During these experiments, the throwing order was randomized (to reduce learning effects) and for each distance the subjects performed 6 throws for a total of 18 throws. A description of the motion and the experimental setup are featured in Figures 1 and 2. The overhead throw is composed of four main stages [26]: starting position, cocking, acceleration, and release and follow-through. In the starting position, the thrower positions his body sideways with respect to the intended target. The cocking phase consists of the motion between the starting position until maximum external rotation is reached before the ball starts to move forward. The acceleration begins as the ball is moved forward and finishes when the ball is released. This phase is known as the explosive phase since the velocity of the ball changes from zero to its maximum in a short time period. Finally, the release and follow-through phase consists in a deceleration of the throwing arm once the ball is released.

For each throw, the subject stood in starting position (see Figure 1). Recording began (onset) when a motion of the hand was detected (threshold at 0.05 m/s) and ended when the ball was released (offset).

#### 2.1.3. Data Acquisition and Processing

During the throwing task, the activity of several muscles of the right arm and body kinematics were recorded. For this study, we focused on studying 4 degrees of freedom (DoF) and 6 muscles. The segmental interaction principle states that energy can be transferred between segments, and in both simultaneous and sequentially coordinated movements, energy is transferred through the linked segment system of the body [28]. However, studies have shown that unlike baseball throwing, in football throwing (or passing), the rotations or contributions of the legs, pelvis, and upper torso are limited [29]. Thus, we decided to first focus on the arm's kinematic chain (beginning at the glenohumeral joint) and on the degrees of freedom with the highest contribution to throwing per segment. These degrees of freedom were the shoulder internal/external rotation and shoulder, elbow, and wrist flexion/extension. The shoulder internal/external rotation and elbow flexion/extension were specially selected due to the fact that they are the major upper limb actions during throwing [23], mainly during the acceleration phase [30].

Next, we selected a set of muscles which contained (1) at least an agonist and antagonist muscle per each DoF under study, (2) muscles with important contributions during throwing [31, 32], and (3) muscles which could be reached by surface EMG electrodes [33]. Thus, the recorded muscles were the deltoid posterior and anterior, the biceps, the triceps long, and the wrist extensors (extensor digitorium, extensor carpi radialis and ulnaris) and flexors (flexor digitorium, flexor carpi radialis and ulnaris), which were recorded as a group.

**Figure 3:**
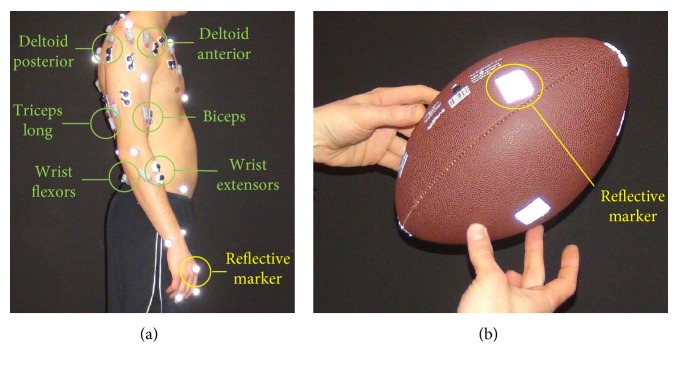
Marker and EMG placement [33, 34]. Recommendations were followed for the EMG placement, whereas reflective markers were placed following the ISB recommendations with small adjustments [38–41].

The muscle activity was collected using wireless surface EMG electrodes (Cometa Waveplus EMG system) and well-known electrode placement protocols [33, 34]. This activity was then processed using a standard protocol [35]: the EMGs were amplified (gain 1000), digitized (1 kHz), band-pass filtered (10–450 Hz, 4th order Butterworth filter with no phase shift), rectified, and low-pass filtered (6 Hz, 4th order Butterworth filter with no phase shift [36]). Additionally, electrocardiogram (ECG) artifacts were removed using an ICA-based filtering procedure [37]. Motion was captured using a Vicon system (16 cameras, 100 Hz sampling rate) and reflective markers. The markers were placed on bony landmarks (49 markers) as recommended by the International Society of Biomechanics (ISB) [38–41], around the target (6 markers), and on the ball (9 markers) (Figure 3). Each of the marker trajectories was low-pass filtered (10 Hz, 4th order Butterworth low-pass filter with no phase shift).

### 2.2. Control Features

The throwing motions can be characterized at three different levels: in the task space, in the joint space, and in the activation space (muscular space). The following sections aim at characterizing the motion at each of these levels through the definition of a set of kinematic and muscular features.

#### 2.2.1. Task Space Features

**Figure 4:**
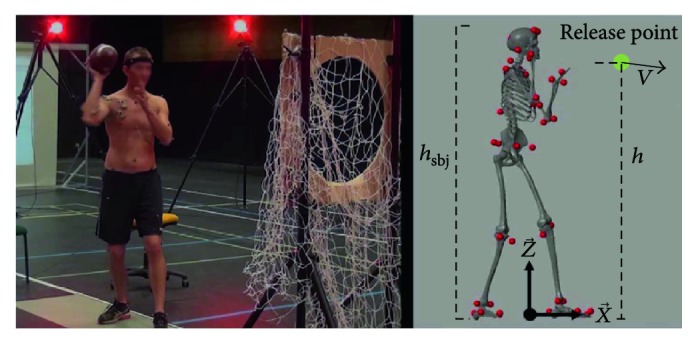
Subject at release time and corresponding task space features. The features consist of the hand release height and the hand velocity of release. Marker occlusion prevented the definition of the angle of release as a reliable feature.

Overhead throwing motions are horizontal projectile motions which are determined by three factors: velocity of release, height of release, and angle of release [42]. Based on this observation, a set of task space features was defined and analyzed across task space conditions. These task features were the hand velocity of release and the hand release height which was normalized by the subject's height, as shown in Figure 4. The angle of release was not considered due to its difficult estimation caused by marker occlusion—some of the markers of the arm were lost at the time of release of the ball, and the release angle computation was very sensitive to the methods used to reconstruct missing trajectories of the markers. Nevertheless, studies have shown that the most important parameter when determining the range of throwing is the release speed [43, 44]. This is also evidenced by the equations of projectile motion, which show that the range is roughly proportional to the square of the release speed.

The time of release (*t*_rel_) was computed as the instant at which maximum hand velocity was reached, since it is known that this event occurs almost in parallel to the ball release in the acceleration phase. For this purpose, a reflective marker was placed on the outer side of the hand (third metacarpal bone) and its position was recorded. After the derivation of this marker's trajectory, the maximum hand velocity or velocity at release (*v*) was computed as follows:

(1) $$\begin{array}{c} v=\max\left(\sqrt{v_{x}{\left(t\right)}^{2}+v_{y}{\left(t\right)}^{2}+v_{z}{\left(t\right)}^{2}}\right), \end{array}$$

where *v*_*x*_(*t*), *v*_*y*_(*t*), and *v*_*z*_(*t*) are the velocity components of the hand marker in the global coordinates frame, as defined in Figure 4 ($\vec{x}$ front, $\vec{z}$ up). The hand height at release (*h*) was determined as follows and divided by the subject's height to allow an intersubject comparison

(2) $$\begin{array}{c} h=\frac{h_{z}\left(t_{rel}\right)}{h_{sbj}}, \end{array}$$

where *h*_*z*_ is the hand marker's position component along z and *h*_*sbj*_ is the height of the subject.

These two features were computed for each subject and repetition. Then, they were grouped per throwing distance (*d*), which could be 2 m, 4 m, or 7 m, yielding a total of six vectors. The means and standard deviations of these vectors were later calculated, resulting in one task feature vector for subject *j*:

(3) $$\begin{array}{c} f_{T,j}=\left[{\bar{v}}_{2m,j}\;{\bar{v}}_{4m,j}\;{\bar{v}}_{7m,j}\;{\bar{h}}_{2m,j}\;{\bar{h}}_{4m,j}\;{\bar{h}}_{7m,j}\right]. \end{array}$$

#### 2.2.2. Joint Space Features

The joint space features consisted of the joint positions and velocities. The joints positions were estimated from motion capture, with an inverse kinematics method allowing the segment lengths and marker positions to be calibrated [45]. The joint velocities where computed by deriving the joint position trajectories. The joint space analysis focused on the following degrees of freedom of the throwing arm: shoulder internal/external rotation and shoulder, elbow, and wrist flexion/extension (*q*_1_(*t*), *q*_2_(*t*), *q*_3_(*t*), and *q*_4_(*t*), resp.).

An average trajectory was computed for each subject, each degree of freedom, and each throwing distance. These trajectories were later time normalized across subjects in order to allow the intersubject comparison in Section 2.3.1. A joint space feature vector per subject *j* was constructed, containing the mean joint position ${\bar{Q}}_{d}$ matrices per throwing distance:

(4) $$\begin{array}{c} f_{Q,j}=\left[\begin{array}{lll} {\bar{Q}}_{2m,j}\left(t\right) & {\bar{Q}}_{4m,j}\left(t\right) & {\bar{Q}}_{7m,j}\left(t\right) \end{array}\right], \end{array}$$

where each joint position matrix contains the average positions $\left(\bar{q}\left(t\right)\right)$ per DoF

(5) $$\begin{array}{c} {\bar{Q}}_{d}=\left[\begin{array}{llll} {\bar{q}}_{1}\left(t\right) & {\bar{q}}_{2}\left(t\right) & {\bar{q}}_{3}\left(t\right) & {\bar{q}}_{4}\left(t\right) \end{array}\right]. \end{array}$$

#### 2.2.3. Activation Space Features

The control done at the muscle level is the one that interests us the most, since it is the actuation space. This control can be described via muscle activations. However, an activation representation is redundant since there are more muscles than degrees of freedom and each muscle needs its own activation signal. A simpler and less redundant representation of these signals can be achieved via muscle synergies [18, 46].

One way to represent such synergies is via the time-invariant synergy model [8, 25]. In this model, a synergy **w**_*i*_ is defined as a **M** × 1 vector of coefficients, specifying the relative activation level of M-muscles. Each synergy is paired with a time-varying combination coefficient vector **c**_*i*_(*t*)(1 × *T*), which determines its temporal evolution. A set of N-synergies can be linearly combined to generate M-muscle activation patterns *A*(*t*):

(6) $$\begin{array}{c} A\left(t\right)=WC\left(t\right)=\left[\begin{array}{llll} w_{1} & w_{2} & \ldots & w_{N} \end{array}\right]\left[\begin{array}{l} c_{1}\left(t\right)\\ c_{2}\left(t\right)\\ \ldots \\ c_{N}\left(t\right) \end{array}\right], \end{array}$$

where *A*(*t*) is the **M** × *T* sample matrix containing the recorded muscle activations patterns, **W** is the **M** × **N** muscle synergy matrix, and **C**(*t*) is the **N** × *T* samples combination coefficient matrix. To separate and highlight the contribution of each synergy **w**_*i*_ and its coefficient **c**_*i*_(*t*) to the muscle activation patterns, the previous equation can also be written as

(7) $$\begin{array}{c} A\left(t\right)=\overset{N}{\underset{i=1}{\sum }}w_{i}c_{i}\left(t\right). \end{array}$$

Based on this model, the time-invariant activation space feature *f*_*A*_ was defined as the matrix **W**, and the time-variant activation space feature *f*_*A*_*t*__(*t*) was defined as the matrix **C**(*t*):

(8) $$\begin{array}{c} f_{A}=W,\\ f_{A_{t}}\left(t\right)=C\left(t\right), \end{array}$$

where

(9) $$\begin{array}{c} C\left(t\right)=\left[\begin{array}{lll} C_{2m}\left(t\right) & C_{4m}\left(t\right) & C_{7m}\left(t\right) \end{array}\right]. \end{array}$$

Each submatrix **C**_*d*_(*t*) is of dimensions **N** × *T*_*d*_, where *T*_*d*_ is the total number of samples contained in the throws to distance *d*.

In our case, this model was used in two methods (see Section 2.3.2). Their results were compared to test their robustness and consistency. The first method consisted of extracting a synergy model (**W**, **C**(*t*)) per subject, and the second method consisted of extracting one synergy model representative of all subjects. Thus, in the first case, a variety of **W** matrices were generated representing each subject's throw, and in the second case, a single **W** matrix was generated representing all subjects and throws.

For both models, the combination coefficient matrix **C**(*t*) encoded the temporal evolution of the synergies during each throw. These coefficients will be further described in terms of (1) their shapes, (2) how their energy changes with throwing distance, and (3) their triggering order. In general, the average image of the energy ${\bar{E}}_{c_{i},d}$ of each combination coefficient **c**_*i*,*d*_ contained in matrix **C**_*d*_(*t*) was computed as follows:

(10) $$\begin{array}{c} {\bar{E}}_{c_{i},d}=\frac{\sum_{s=1}^{T_{d}}{\left|c_{i,d}\left(t_{s}\right)\right|}^{2}}{n}, \end{array}$$

where *n* is the number of trials per throwing distance and *t*_*s*_ the current time sample.

### 2.3. Control Representation Extraction

Once the features to analyze were defined at each level (task, joint, and activation spaces) and for each subject *j*, control representations based on these features were extracted. The objective of such an extraction was to verify if a generic control representation existed for overhead throwing across subjects. Such representations are denoted by an index *All*, which generalizes the subject feature vectors (*j*) of the previous sections to all participants.

The identification of these representations was made through clustering algorithms for the time-invariant features and averaging and cross-correlation for the time-variant features.

Clustering is a technique that consists of the assignment of features into groups or subsets based on similarity criteria. In the next sections, we will see that the existence of a generic representation in each space will depend on the number of clusters or groups found with these techniques.

The first step before using the clustering algorithms is feature scaling. This preprocessing step is necessary due to the fact that clustering algorithms use distances to classify features. Thus, features should be standardized such that they have contributions of equal importance in the distance measurements.

Two different types of clustering algorithms were used to extract control representations from the time-invariant features: a centroid-based clustering (*k*-means) algorithm and a connectivity-based clustering algorithm (hierarchical clustering). These two algorithms were used in order to verify if different techniques yielded similar control representations. Furthermore, the specific interest in using hierarchical clustering was to verify if the chosen number of clusters of the *k*-means algorithm matched the natural divisions in the data.

*K*-means clustering is an iterative algorithm for data partitioning that assigns or classifies features into one of *k* clusters defined by centroids. The main steps of the algorithm are the following, given *k*: (1) select *k* initial cluster centroids, (2) compute the distances between each feature to each cluster centroid, (3) assign the features to the cluster with the closest centroid all at once (phase 1), and individually reassign points if it reduces the sum of distances (phase 2), (4) obtain new centroids by averaging the features in each cluster, and (5) repeat steps 2–4 until the assignments do not change or the iterations reach their maximum. The *k*-means++ algorithm in MATLAB was used with a squared Euclidean norm to compute distances. The advantage of this algorithm is that it uses the heuristic in [47] to find centroid seeds for *k*-means clustering. This induces a faster convergence to higher quality solutions or to a lower sum-of-squares point-to-cluster centroid distances (within each cluster). Finally, in order to assess the *k*-means clustering quality, an indicator called cluster silhouette was computed [48, 49]. This indicator enables us to distinguish clear-cut clusters from weak ones. It measures how similar the features are to features in their own cluster, when compared to features in other clusters, and is computed as follows:

(11) $$\begin{array}{c} Sil=\frac{\left(b_{j}-g_{j}\right)}{\max\left(b_{j},g_{j}\right)}, \end{array}$$

where *g*_*j*_ is the average distance from the *j*th feature to the other features in the same cluster as *j* and *b*_*j*_ is the minimum average distance from the *j*th point to points in a different cluster, minimized over clusters. The silhouette value can range from −1 to 1. By averaging the silhouette values of each feature in the cluster, an average silhouette $\left(\bar{Sil}\right)$ can be obtained for the entire cluster. A subjective interpretation for this value was proposed by the authors of [49] to assess the clustering quality, as shown in Table 1.

This interpretation was used to select with which number *k* of clusters the data was well separated $\left(\bar{Sil}\geq 0.71\right)$ or if no separations could be made, in which case only a single cluster exists.

To complete this assessment, hierarchical clustering was also used to partition the feature space into groups. Hierarchical clustering is an algorithm for cluster analysis that aims at grouping features at different levels using a cluster tree or dendrogram. In agglomerative hierarchical clustering, each feature starts in its own cluster; these clusters are then combined via a metric and a linkage criterion. The metric defines a distance between pairs of features, and the linkage criterion defines the distance between sets by computing the pairwise distances between features. An advantage of this strategy is that it does not need an initial indication of the number of clusters, and therefore, it reveals the natural divisions in the data. For its implementation, the hierarchical algorithm tools in MATLAB were used with the Euclidean distance as metric and an unweighted average distance (Euclidean) for the linkage.

The following sections present how these methods and the synergy extractions [9] were used to extract control representations across subjects.

#### 2.3.1. Task and Joint Space Control Representation Extraction

First, we determined if a common representation existed across subjects in task space. Thus, the feature vectors **f**_*T*,*j*_ in (3) were first standardized and then given as inputs to the clustering algorithms. First, the *k*-means algorithm was applied by varying the number of clusters and checking how well the clusters were separated thanks to the clusters' silhouette values. In this space, since each subject is characterized by a single vector, we expect a common representation to exist when *k* = 1. In other words, when the features are so similar, that well-separated clusters cannot be formed. If this was the case, then, the common strategy was defined by averaging the task feature vectors across subjects:

(12) $$\begin{array}{c} f_{T,All}=\left[\begin{array}{llllll} {\bar{v}}_{2m,All} & {\bar{v}}_{4m,All} & {\bar{v}}_{7m,All} & {\bar{h}}_{2m,All} & {\bar{h}}_{4m,All} & {\bar{h}}_{7m,All} \end{array}\right]. \end{array}$$

To further verify the results of *k*-means, hierarchical clustering was then applied. This algorithm does not need an initial estimate of the desired number of clusters; thus, it was used in order to determine if the natural cluster divisions of the data agreed with the results provided by *k*-means. In other words, if no natural cluster divisions were found and a common task control strategy across subjects existed. Finally, a Wilcoxon rank sum test was performed on the features across subjects to detect significant changes in their values with regard to the throwing distance (confidence level below 0.05).

At the joint space level, the features **f**_*Q*,*j*_ used to represent the motion are all time varying (average joint positions per subject). Therefore, cross-correlation was used to evaluate the similarity of the joint trajectories and velocities across subjects and throwing distances. A high correlation signified that the joint trajectories or velocities were similar among subjects. Low correlations signified very low kinematic similarities. The common joint space strategy was then defined by averaging the joint trajectories and velocities across subjects:

(13) $$\begin{array}{c} f_{Q,All}=\left[\begin{array}{lll} {\bar{Q}}_{2m,All}\left(t\right) & {\bar{Q}}_{4m,All}\left(t\right) & {\bar{Q}}_{7m,All}\left(t\right) \end{array}\right]. \end{array}$$

#### 2.3.2. Activation Space Control Representation Extraction

The synergies and their combination coefficients (Section 2.2.3) were extracted via a NMF (nonnegative matrix factorization) [50] algorithm. This algorithm decomposes a nonnegative matrix into a nonnegative linear combination of basis vectors, by solving the following optimization problem:

(14) $$\begin{array}{c} \begin{array}{ll} \underset{W,C}{\mathrm{minimize}} & \frac{1}{2}{\left\|A\left(t\right)-WC\left(t\right)\right\|}_{F}^{2},\\ \text{subject to} & W,C\left(t\right)\geq 0. \end{array} \end{array}$$

When applying this algorithm, the synergy model order or number of synergies to extract should be defined. To do this, we used two criteria. The first criteria consisted of choosing a number of synergies *N* less than the number of recorded muscles *M* = 6 in order to obtain a lower dimensional control representation. The second criteria attempted to preserve a good quality in the reconstruction of the original activations. Therefore, a criterion based on the average coefficient of determination *r*^2^ between the original and reconstructed muscle patterns [9, 18] was used. This criterion states that the chosen number of synergies should correspond to the sharpest change in the slope of the *r*^2^ curve (as in the submitted version within the results). This change in slope is interpreted as the point separating “structured” from noise-dependent variability. After this point, additional synergies start to capture only the small residual noise-dependent variability; therefore, this can be used to define the minimum number of synergies that capture the task-related features [46, 51, 52]. We highlight the fact that these criteria guarantee that the number of control variables *N* will be less than the number of muscles or actuators; however, there is no guarantee that they will be less than the number of DoF. This is a possible added value of a representation through synergies. The NMF algorithm used was the one developed in [53] and the update rule used was the nonnegative least squares one.

We employed two methods for identifying a representative synergy (or time-invariant features) using this extraction algorithm. The first is based on *k*-means [54] and hierarchical clustering, and the second one is based on the identification procedure in [9]. The comparison of the results extracted from both methods was useful to test the consistency and robustness of these extraction methodologies.

The first method consisted of 3 stages: (1) extraction of individual subject synergy models, (2) standardization of **w**_*i*_ vectors, and (3) application of *k*-means and hierarchical clustering algorithms. In the first stage matrix, *A*(*t*) (6 muscles × 3600 samples) was constructed by concatenating the activation signals for all the trials of individual subjects. This method enabled us to take into account intrasubject variability in the synergy extraction. The concatenated signals were normalized by their maximal value to obtain activations framed between 0 and 1. Next, NMF was applied on this matrix to obtain one N-synergy model (**W**, **C**(*t*)) per subject. Once a model was obtained for each subject, the synergy matrices **W** were standardized for their use in the clustering algorithms. Essentially, each synergy **w**_*i*_ of each subject was a feature vector containing the relative activation levels of the muscles. These vectors were treated individually and without specifying their correspondence to a specific subject. They were used to create a synergy pool on which *k*-means and hierarchical clustering were applied in order to identify common features among this synergy pool. The *k*-means algorithm was applied first while varying the number of clusters *k*. We expected a unique strategy to exist when *k* = *N*, in other words when the number of clusters is equal to the number of synergies extracted for each subject. If this was the case, then the centroids of these clusters represented the mean synergy vectors or the representative activation control representation for all subjects through method I (**W**_I,All_).

(15) $$\begin{array}{c} f_{A,All}=W_{I,All}. \end{array}$$

Finally, hierarchical clustering was applied. This algorithm was used in order to determine if the natural cluster divisions of the data corresponded to the number of *k*-means centroids.

The second synergy extraction method consisted in the direct identification of a common activation control strategy for all subjects, based on [9]. In this method, the NMF algorithm was applied on a matrix *A*(*t*) (6 muscles × 36,000 samples), constructed by concatenating the activation signals for all trials of all subjects. Therefore, by applying NMF on this pool of EMG signals, one common synergy model (**W**_II,All_) was found for all subjects.

(16) $$\begin{array}{c} f_{A,All}=W_{II,All}. \end{array}$$

However, the coefficients (**C**_II_(*t*)) in this method encoded how much and when each synergy was triggered for each repetition and subject. Therefore, to identify a common time-varying control representation for all subjects and repetitions $\left({\bar{C}}_{II,All}\left(t\right)\right)$, averaging and correlation computation were used. First, the mean combination coefficients per subject per throwing distance were computed. Next, cross-correlation was used to make comparisons across subjects at each throwing distance. Thus, a common combination coefficient was computed by making a second averaging across all subjects.

(17) $$\begin{array}{c} f_{A_{t},All}\left(t\right)={\bar{C}}_{II,All}\left(t\right), \end{array}$$

where ${\bar{C}}_{II,All}\left(t\right)$ contains the coefficient matrices per throwing distance

(18) $$\begin{array}{c} {\bar{C}}_{II,All}\left(t\right)=\left[\begin{array}{lll} {\bar{C}}_{II,2\;m,All}\left(t\right) & {\bar{C}}_{II,4\;m,All}\left(t\right) & {\bar{C}}_{II,7\;m,All}\left(t\right) \end{array}\right]. \end{array}$$

## 3. Results and Discussion

### 3.1. Global Considerations

The motion, as defined in the task description above, had an average duration of 1.67 ± 0.27 s for all the throws made by all the subjects. Thus, the standard deviation seemed sufficiently low to compare the different throws and normalize them against time, as it has been done for some of the processing of extraction. The subjects had a global performance higher than 80%, meaning that the task was quite easy to perform and reproducible from one trial to one other. The following sections detail the representations extracted from the experimental data in the task, joint, and activation spaces. For all the cross-correlation we performed, the mean value of the lag was about less than 10^−15^% of the signal length, meaning that most of the signal shapes were comparable directly. Therefore, we did not present the lags related to cross-correlation results in the corresponding tables.

### 3.2. Task Space Control Representation

**Figure 5:**
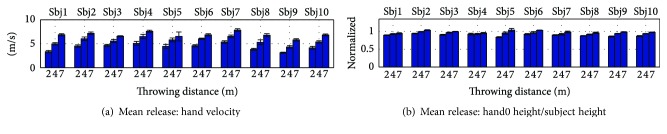
Task space features per subject and throwing distance. Both hand release velocity and release height increased with the throwing distance for all the subjects.

The subject task features **f**_*T*,*j*_ were collected and a representative task space control representation **f**_*T*,All_ was extracted as described in Section 2.3.1. The task feature vector for each subject is featured in Figure 5. A gradual increase in hand release velocity and height can be seen across subjects as the throwing distance increases. Moreover, as evidenced by the Wilcoxon rank sum test, this increment is statistically relevant in 9/10 subjects for the hand velocity and in 7/10 subjects for the hand height.

Next, *k*-means and hierarchical clustering were applied on the feature vectors in order to determine if one sole task space control representation existed. We expected a unique control representation if no strong separated groups can be found among the subject task vectors, in other words if a single cluster exists.

**Figure 6:**
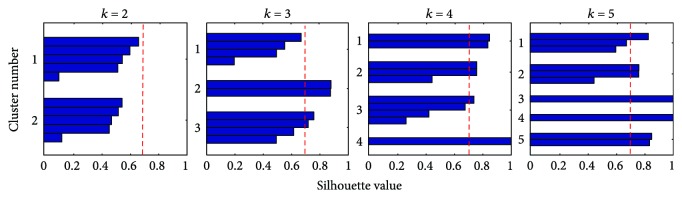
Task space cluster separation quality using *k*-means. Strongly separated clusters $\left(\bar{Sil}\geq 0.71\right)$ containing a similar number of subjects cannot be found. A single cluster exists across subjects.

*k*-means was first applied while varying the number of clusters *k*.  Figure 6 shows each cluster's silhouette. The average silhouette value $\bar{Sil}$ is below 0.71 (Table 1) as *k* increases. At *k* = 5, it reaches a value above this threshold but clusters containing 1-2 subject vectors begin to be formed. Therefore, since the *k*-means analysis did not differentiate the subjects, a common representation of the control at the task space level can be obtained from the averaging of the task space features.

**Figure 7:**
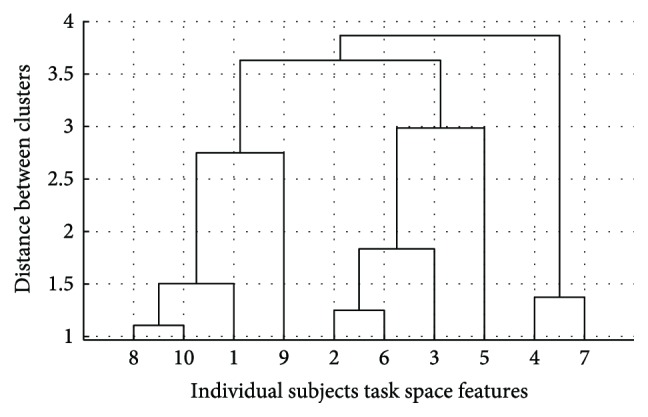
Task space cluster separation using hierarchical clustering. In this case, the clusters are not well separated.

**Figure 8:**
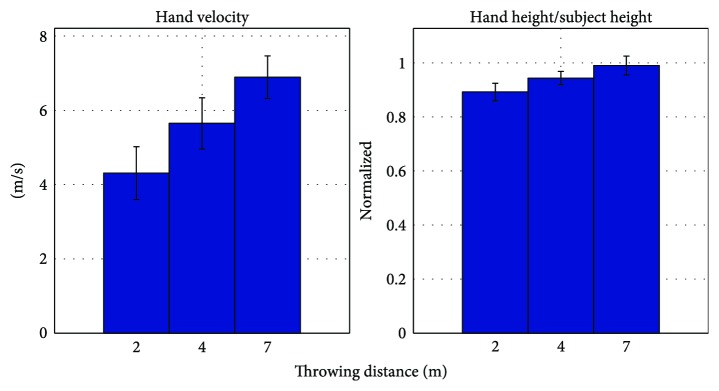
Representative task space representation for all subjects (**f**_*T*,All_). These are the mean and standard deviation values of the features shown in Figure 5. The global increase of both features with regard to the throwing distance is straightforward.

Hierarchical clustering was then applied in order to verify if the results of the *k*-means clustering were consistent with the natural division of the data. The hierarchical clustering was only used to consider qualitative and visual informations about data division.  Figure 7 features the resulting cluster tree. In this tree, no visually significant divisions are found. This is shown by the fact that heights of the links at each level are not qualitatively different from the heights of the links below them, indicating a high closeness across groups. Furthermore, with this procedure, we can see that as the number of clusters increases, groups containing very few subject vectors begin to be formed. Consequently, we concluded that all subjects were presenting similar changes in the task space features with regard to the task constraints (throwing distance). In other words, subjects increased significantly the hand release velocity and height as the throwing distance increased. The average task features **f**_*T*,All_ were computed by averaging the subject task feature vectors and it is shown in Figure 8. Velocity increments of about 1.3 m/s and hand-height/subject-height increments of 0.05 are seen as the distance increases by 2 to 3 m. Moreover, the range is roughly proportional to the square of the release speed, as can also be evidenced through the equations of projectile motion. Thus, our results are consistent with other studies that indicate an increase in height and speed with throwing distance and the existence of a proportionality relationship between speed and range [23]. This is a straightforward result that may be mostly induced by the motion constraints (distance to throw, motion type) and the difference of strategy between subjects may appear in the amount of changes from one distance to one other as it can be observed in Figure 5. However, the averaging of the task space features as a unique representation of the control in the task space makes sense since the trends featured in Figure 8 respect the same pattern as the one seen for all the subjects.

### 3.3. Joint Space Control Representation

The subject joint features **f**_*Q*,*j*_ were then used to determine if a common joint space control representation **f**_*Q*,All_ existed, as described in Section 2.3.1. The features showed a high repeatability across subjects at each throwing distance, regardless of the inexperience and small differences in style of our throwers. These kinematic similarities were quantified as correlations among subjects and are shown in Table 2. As the throw is performed, the motion is repeatable in the forward direction. Thus, high correlations are seen in the joint trajectories, especially in the shoulder (*q*_2_(*t*)) and elbow (*q*_3_(*t*)) flexion/extension. Lower but still significant correlation is seen in the shoulder internal/external rotation (*q*_1_(*t*)) and wrist flexion/extension (*q*_4_(*t*)). The differences in internal/external rotation (*q*_1_(*t*)) could be due to each subject's throwing style, while the differences in wrist flexion/extension (*q*_4_(*t*)) could be linked to the fact that the most distal segments have larger contributions to accuracy over speed [32, 55].

In terms of articular velocities, the motion is less repeatable. Nevertheless, as seen in the previous section, different velocity control strategies in the joint space can result in a common velocity feature in the task space across subjects. These differences may be linked to individual differences in the throwing strategy and cannot be used as a common feature of the control representation in the joint space.

**Figure 9:**
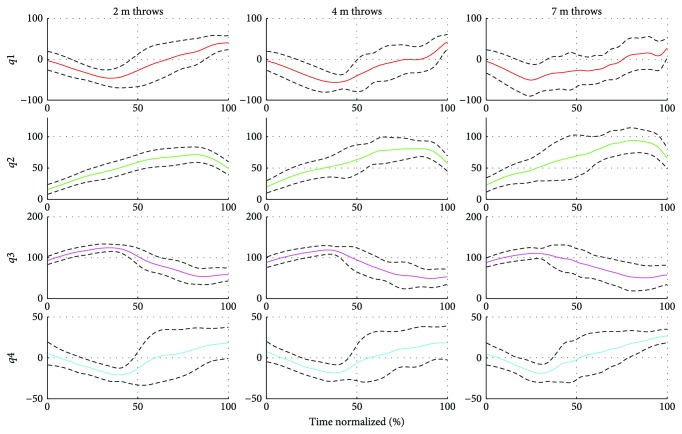
Representative joint space strategy for all subjects (**f**_*Q*,All_). Computed by averaging joint features across subjects for each DoF. Solid lines are the average values and dashed lines the standard deviation values. The joint space analysis focused on the following degrees of freedom of the throwing arm: shoulder internal/external rotation and shoulder, elbow, and wrist flexion/extension (*q*_1_(*t*), *q*_2_(*t*), *q*_3_(*t*), and *q*_4_(*t*), resp.). Angles are given in degrees.

Finally, a representative joint space control strategy was computed by making averages across subjects and throwing distances for joint trajectory included as a feature. This control representation is featured in Figure 9 and in Table 3. Similar kinematic trends are shared across throw types. For instance, as the throw progresses, the shoulder is internally rotated and flexed, while the elbow is extended and the wrist is gradually flexed. Lastly, these similarities were also reflected in the intersubject and interdistance correlation, which resulted in very high correlation coefficients for all DoF, as shown in Table 3.

### 3.4. Activation Space Control Representation

**Figure 10:**
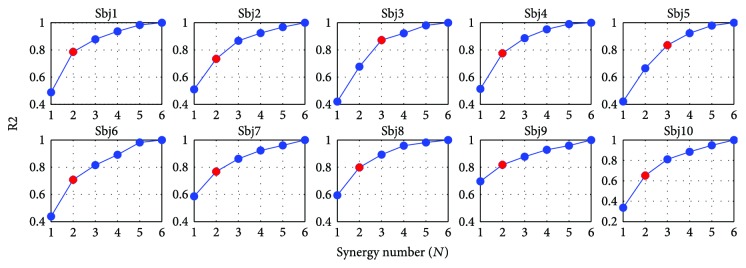
Activation reconstruction quality across synergy models per subject. The NMF algorithm was applied on each of the subject's EMG data set while varying the number of synergies (*N*). The resulting curve depicts the *r*^2^ values for each model.

The synergy extraction method described in Section 2.3.2 was applied on each of the subject's EMG dataset while varying the number of synergies. The objective was to identify a model with less synergies than the number of recorded muscles or actuators (*N* < *M*), for each subject, that would guarantee a good reconstruction of the original EMG signals.  Figure 10 depicts the quality of reconstruction *r*^2^ for each subject and synergy model. The sharpest change in slope of this curve occurred at *N* = 2 for 8 subjects and at *N* = 3 for 2 subjects. Thus, we opted for the 2-synergy model which allowed an average quality reconstruction of 0.7382 across subjects.

#### 3.4.1. Synergy Model (**W**)

**Figure 11:**
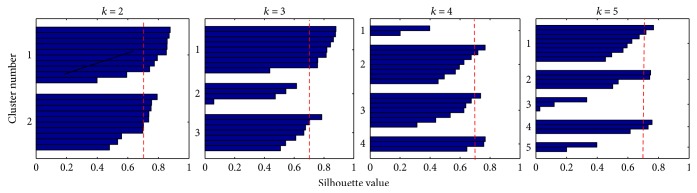
Cluster separation quality using *k*-means. Strongly separated clusters $\left(\bar{Sil}>0.71\right)$ are found at *k* = *N* or *k* = 2. A common activation space control representation exists across subjects.

Method I was applied in order to determine a common representation of the control in the activation space. First, the 2-synergy models were extracted for each subject. Then, a pool containing the individual synergies **w**_*i*_ of all subjects was constructed, without specifying if the synergies belonged to the same subject. Thus, the pool contained 20 synergies (2 synergies per subject). Finally, k-means clustering was applied on this pool while varying the number of clusters *k*. We expected a common control representation to exist when *k* = *N* or when the number of clusters is equal to the number of synergies extracted per each subject.  Figure 11 shows that indeed the best cluster separation is achieved at *k* = *N* or *k* = 2, where the average silhouette value for both clusters is equal to 0.7181. If a higher number of clusters or separations is found, the average silhouette values decrease and clusters containing very few synergies are formed. This evidences that 2 clusters are sufficient to classify the synergies.

**Figure 12:**
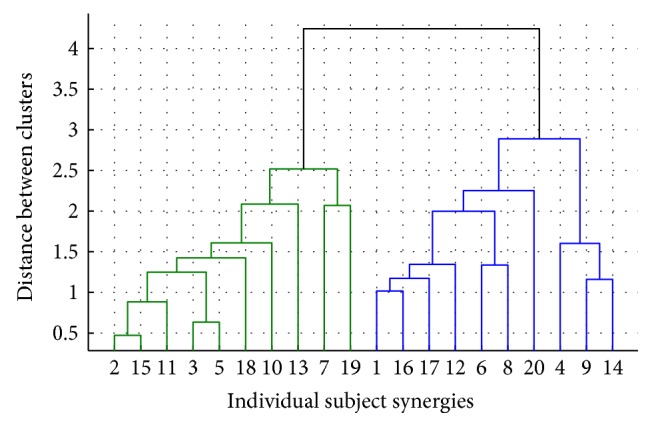
Synergy clusters using hierarchical clustering. Natural data divisions are found when the height of a link strongly differs to the height of the links below it. Thus, in this case, the clusters are well separated.

To further verify if the natural divisions of the data corresponded to 2 groups of distinctive synergies, hierarchical clustering was applied. This resulted in the cluster tree in Figure 12. In this tree, we can see how the 20 synergies in the pool are partitioned into 2 clusters as well. This is shown by the fact that the link separating the synergy data into two branches is inconsistent with the links below it. It indicates a higher closeness among the synergies within each group than across each group.

Interestingly, the individual synergies within each cluster in the tree matched those in the clusters computed via k-means. Thus, a mean activation control representation **W**_I,All_ for all subjects was extracted from the centroids of the 2-cluster model obtained via *k*-means (Figure 6, top). Each of these centroids or mean synergies contains the relative action levels of groups of muscles. Finally, we wanted to demonstrate how well the synergy **W**_I,All_ represented all of the subjects' individual synergies. In order to do this, the normalized dot product between the synergy **W**_I,All_ (centroid) and each of the subjects' 2-synergy models **W** (cluster points) was computed. The results showed that a high similarity exists between these models, with a mean normalized dot product of 0.9495 ± 0.0485 for **w**_1_ and 0.9170 ± 0.0537 for **w**_2_.

Method II was then applied to identify the representative synergy model directly from a pool containing the EMG signals of all subjects. Thus, this pool contained 6 signals (one per muscle), and each signal contained 180 concatenated activations corresponding to each of the subjects' trials (10 subjects, 3 throwing distances, and 6 trials per distance). As in the individual subject synergy extractions, the number of synergies was chosen as the number corresponding to the sharpest change in the *r*^2^ curve. This change occurred again at *N* = 2 synergies, where the quality of reconstruction was of 0.6526. This slight decrease in quality of reconstruction with respect to the individual extractions is expected since method II attempts to reconstruct a higher number of trials performed by different subjects simultaneously.

**Figure 13:**
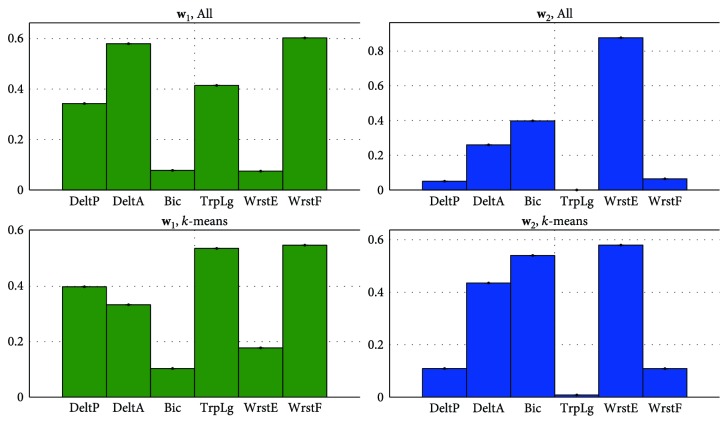
Representative activation space strategy (synergies) **W**_I,All_ and **W**_II,All_ for all subjects. A common control representation in the activation space was identified for the time-invariant part of the synergies (**W**) through method I and method II.

The resulting representative synergy **W**_II,All_ is depicted in Figure 13. Again, each synergy contains the relative activation levels of a group of muscles throughout the motion. The first synergy **w**_1_ can be seen as the agonist synergy, and the second synergy **w**_2_ can be seen as the antagonist synergy to the motion. Therefore, **w**_1_ contains a high activation of muscles corresponding to shoulder flexion, internal rotation (deltoid anterior), elbow extension (triceps longs), and wrist flexion (wrist flexor group). While **w**_2_ contains a high activation of muscles corresponding to elbow flexion (biceps), wrist extension (wrist extensor group), and a very low activation of the shoulder muscles (deltoid anterior and posterior).

Finally, the representative synergy vectors (**W**) computed with both methods are similar, as shown by their normalized dot products (0.9248 for **w**_1_ and 0.9524 for **w**_2_). Consequently, a common grouping and relative activation of muscles were found for different task space conditions and subjects during a throwing motion. This emphasizes the consistency of the results obtained by both methods to find a proper activation space control representation of the motion. However, in order to define a common control representation for throwing in the activation space, it is also necessary to identify a representative pattern for the time-varying part of the synergies (combination coefficients). The following section presents the results of this analysis.

#### 3.4.2. Combination Coefficients (C)

**Figure 14:**
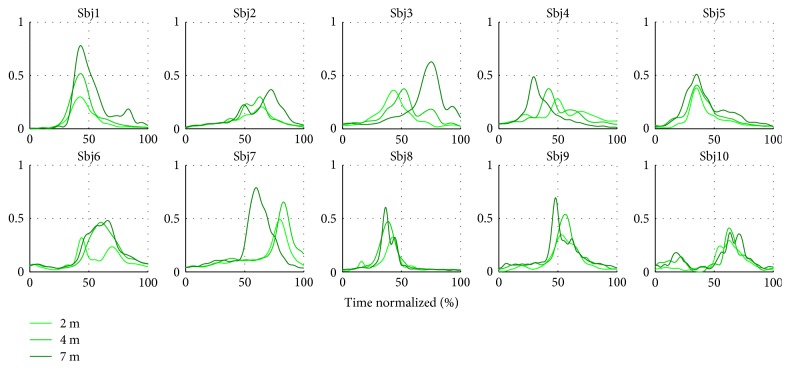
Average combination coefficient *c*_1_ per subject and throwing distance.

**Figure 15:**
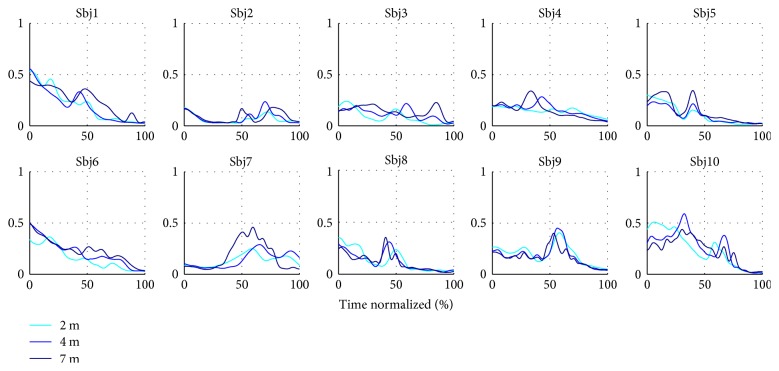
Average combination coefficient *c*_2_ per subject and throwing distance.

Method II also resulted in a set of time-varying coefficients which encoded the triggering times and intensity for each subject and their repetitions *C*_II_(*t*). The average coefficients computed per subject and throwing distance are featured in Figures 14 and 15. Repeatable trends can be seen among and across subjects. For instance, the first coefficient *c*_1_ is generally bell shaped (as the velocity profile in ballistic movements), while the second coefficient *c*_2_ is more irregular, it has a lower amplitude, and it tends to decrease as the throw is performed. A considerable intersubject repeatability at each throwing distance is also demonstrated by high correlation coefficients, as featured in Table 4.

**Figure 16:**
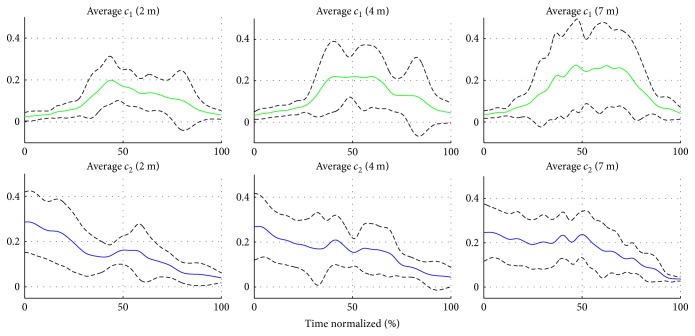
Representative activation space strategy (combination coefficients) ${\bar{C}}_{II,All}\left(t\right)$ for all subjects. A common control strategy in the activation space was identified for the time-variant part of the synergies through method II.

The high intra- and intersubject repeatability outlines the existence of similar patterns of activation for each synergy across subjects and throwing distances. Therefore, an activation space control representation ${\bar{C}}_{II,All}\left(t\right)$ was computed by performing averages across subjects at each throwing distance. The mean coefficients per distance are depicted in Figure 16. A high interdistance correlation is seen for both coefficients (Table 5). Thus, these coefficients not only preserve the main trends in each of the subjects' averages but also emphasize the similarities in terms of shape across throwing distances.

**Figure 17:**
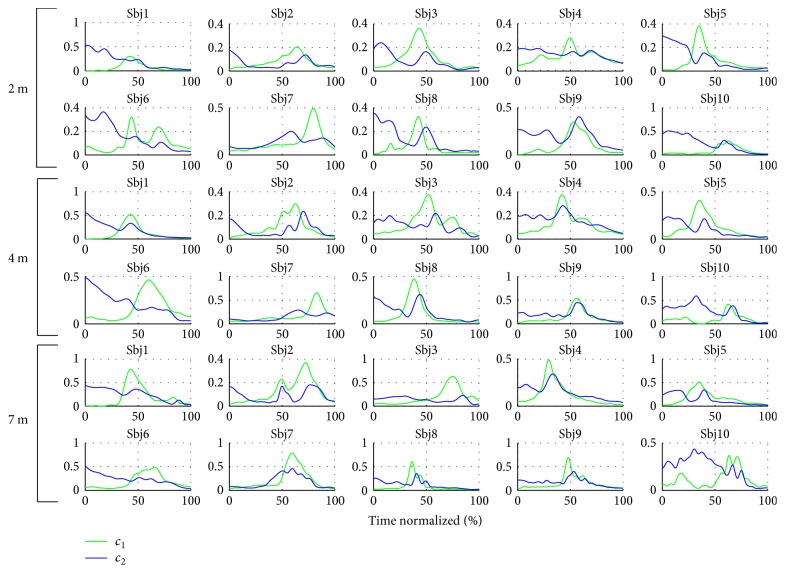
Triggering order and coactivation per subject and throwing distance. A repeatable triggering tendency is seen across subjects: (1) *c*_2_ triggering, (2) *c*_1_ triggering, and (3) *c*_2_ triggering. This sequence is consistent with the expected triggering in ballistic motions.

Besides a repeatability in terms of shape, the combination coefficients exhibit discrepancies across subjects.  Figure 17 shows the coefficients *c*_1_ and *c*_2_ from Figures 14 and 15 in one same plot. Globally, at the beginning of the throw *c*_2_ (antagonist synergy) is activated, then, the amplitude of this synergy is diminished, until *c*_1_ (agonist synergy) is activated. At this moment *c*_2_ is activated again, and the most significant coactivation occurs among the synergies. The same behavior is seen on the representative activation space strategy in Figure 16. This is consistent with the fact that ballistic movements exhibit concurrent agonist and antagonist muscle activation [56]. During these motions, a first activation is needed to accelerate the limb toward the target (*c*_1_), followed by a second activation to decelerate and stop the movement (*c*_2_). This sequence of bursts (from antagonist to agonist and from agonist to antagonist) is a characteristic of the antagonist activity in the upper extremity while throwing. Such “triad” burst sequences have been previously identified in EMG analysis of throwing (at the wrist and elbow muscles) [32] and in badminton smash strokes [55].

Individual differences in combination coefficients triggering can be seen between subjects, especially for *c*_1_. This indicates that even if it is possible to find a common representation of the control in the activation space for time-invariant features **W**, the combination coefficients *C* encapsulate individual strategies and differences between subjects.

**Figure 18:**
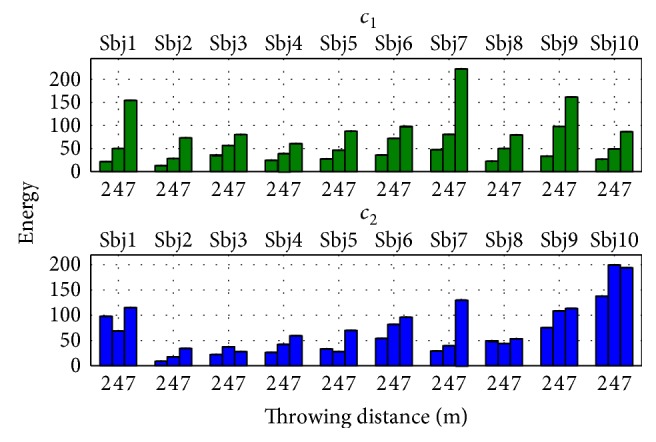
Combination coefficient energy per subject and throwing distance. The energy in *c*_1_ (agonist synergy) gradually increases with throwing distance.

Another characteristic that was analyzed was the change in energy across throw types.  Figure 18 shows the average energy at each throwing distance per subject, as described in 10. The results show that the energy changes in the coefficients are linked to changes in the task space features: like the task space features, the energy in the coefficients increases with the throwing distance. For *c*_1_ (agonist synergy), this increment is always gradually incrementing, and it is statistically relevant for 6/10 of the subjects. This increment in the actuation signals (or synergies) is consistent with the increment in torque magnitudes, observed during the synthesis of throwing motions to different ranges [23].

This link between the task space and the activation space is fundamental in order to specify muscle-based controllers available to synthesize motions from task space goals. Indeed, such a controller will define a control law to actuate the muscle in order to achieve task space goals and the results of the current study are helpful to design these control laws [2].

#### 3.4.3. Activation Reconstruction

**Figure 19:**
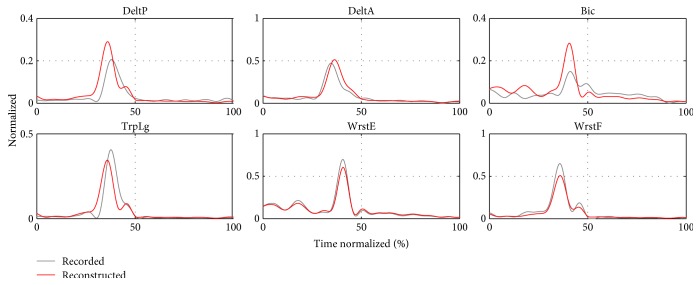
Example 1: activation reconstruction using **W**_II,All_ and **C**_II,All_(*t*).

**Figure 20:**
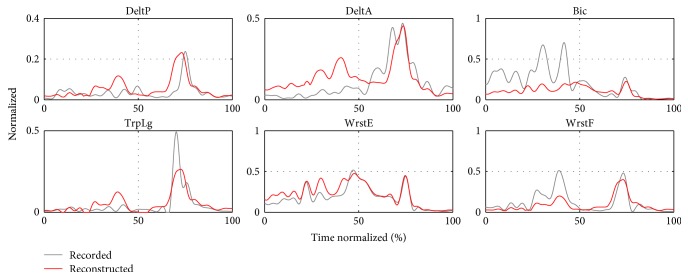
Example 2: activation reconstruction using **W**_II,All_ and **C**_II,All_(*t*).

Finally, we show the quality of EMG reconstruction using the representative synergy model (**W**_II,All_, **C**_II,All_(*t*)) found through method II. We finally get an overall reconstruction quality of *r*^2^ = 0.6526 for the 180 concatenated muscle activations. This is reflected through different degrees of quality reconstruction among the subject trials. In Figures 19 and 20, examples of the activation reconstruction of a 7 m trial for different subjects are shown. In the first case, the triggering order and shape of the reconstructed activations follow closely the recorded ones. In the second example, the original activations contain many small oscillations, which are not well reconstructed. These oscillations may be noise artifacts and were therefore excluded from the reconstruction by the reconstruction quality criteria *r*^2^, as it has been explained in the Methods section. Moreover, considering the number of trials that are being reconstructed simultaneously, such differences in reconstruction accuracy were expected.

**Figure 21:**
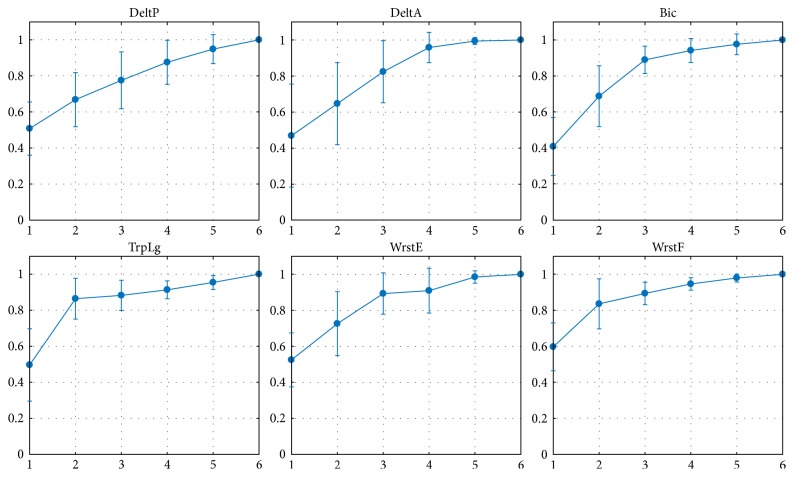
Activation reconstruction quality per muscle. Results per muscle show discrepancies in the reproducibility of the results. Some muscles have relatively consistent patterns to reconstruct with 2 synergies (triceps long, wrist flexor group) whereas some other may be better reconstructed thanks to higher synergy levels (biceps).

In addition, we can see (Figure 21) the reconstruction quality per muscle with regard to the number of synergies extracted on the global set (method II). We can see that the results are quite consistent from one muscle to one other. Indeed, most muscles respect the rule that the biggest change of slope of *r*^2^ appears after 2 synergies. However, the biceps exhibit a relatively low reconstruction level with 2 synergies and seem to have its highest change in slope at 3 synergies. This result can be explained by the relatively low level of activation of the biceps during the task, that may be less well captured by the synergy extraction than the more activated muscles like the triceps long. In a more general manner, muscles that stabilize the motion may be less well captured by the low-order synergies than the muscles producing the motion.

### 3.5. Summary

The previous results show the existence of a common control representation (for a subset of muscles and DoF) in various throwing tasks, and subjects with no particular training on throwing motions or throwing sports. This representation was described through a set of features in the task, joint, and activation spaces. The control representation identified in the task space consisted of increasing the hand release height and velocity to reach longer distance targets. These endpoint features were achieved through a common set of joint trends, but with different velocity trends across subjects.

In the activation space, a lower dimensional control representation and its link with changes in the task space features were identified. This control strategy consisted of using only 2 synergies (an agonist and an antagonist synergy) to represent the activation of 6 muscles of the right arm. These synergies were triggered with the order and concurrency expected from ballistic movements, and their triggering intensity was linked to the desired launch distance, the increments in velocity, and height of release. Therefore, at the actuation level, we were able to extract a reduced control representation (muscle synergies) linked to task conditions, for a highly redundant, nonlinear, and dynamic motion. Such a method, by providing a compact representation, has the interest to depict the individual and common control features in the way the motion is generated by each subject and seems useful to better understand the control strategies used. This does not prove the existence of a motor control mechanism that would be muscle synergies. However, the results are compatible with the notion of muscle synergies organized by the nervous system to implement such control strategies.

Moreover, the direct extraction of a single synergy model from an experiment involving such a complex motion, and a variety of human morphologies, skills, and task conditions, is also a contribution. The results obtained by both methods of synergy extraction showed encouraging results, since their consistency and robustness was clearly established through their comparison. The accuracy of this synergy model is supported by studies [57] that evidence a higher performance of matrix factorization algorithms in experimental protocols that incorporate unconstrained tasks, a variety of conditions, and motor variability (synergy extraction from EMG time series data and not averages).

It is worth noting that these results span a limited set of degrees of freedom and muscles and that the extracted synergies for this task can change depending on the number and choice of muscles [58]. They also highlight generic but basic mechanisms needed to control an overhead throwing motion to a specific distance. To analyze the accuracy, efficiency, or performance of the throw, studies with additional features at key moments and their relationship to successful target hits are needed. These features could include task space features, such as release angle; joint space features, such as velocities and accelerations at release; and activation space features that include more muscles and quantify subtle difference in the way in which the synergies are triggered across different throws. With more features, we could expect to find more links across task, joint, and activation spaces.

Future contributions could include repeating this analysis on professional throwers (such as football players or pitchers). We expect a higher repeatability at all levels for trained subjects. Also, future analysis could include throws to larger distances and the usage of balls of different masses and sizes.

Finally, the synergies obtained in the current studies will be applied and validated in the domain of muscle-based character animation. For instance, the relationships between the 2 control variables (or synergies) and well-defined task space goals (desired release speed and height) will be exploited to control highly redundant characters. A previous study [8, 24] has already tested synergies on a subject-scaled character. It would be interesting to test the generic synergies presented in this paper on a variety of morphologies. Ultimately, this application could also entail the construction of a synergy database for animation. A database containing synergies and their relationships with task space goals, for a richer variety of motions (reaching, writing, or other arm gestures), degrees of freedom, and muscles, which could also serve as a basis to synthesize motions in physics-based animation.

## 4. Conclusion

It seems that motion control can be encapsulated through lower dimensional control representation of each task we perform, to achieve fast, efficient, and coordinated movements. Synergies encode a variety of muscle information in a reduced set of temporal and spatial signals and are thus a good candidate to represent the control in a compact way. Many studies have extracted muscle synergies from EMG signals in both upper-body and lower-body motions. Our study has found common control features among subjects in the task, joint, and activation spaces, especially through the extraction of muscle synergies from a set of EMG signals, for a dynamic and acyclic motion. A motion which was performed by unexperienced subjects while following general guidelines that allowed a free throwing motion.

We first described the throwing task and experiments from which the control strategy was extracted. Next, we characterized the motion through a set of control features in the task, joint, and activation spaces and detailed the methods to extract them. Finally, the results showed that with this set of features (1) a common control representation exists across subjects, (2) this representation significantly reduces the redundancy in the activation space through the encapsulation of the coactivated muscles in a low-dimensional representation (2 synergies encode the actions of 6 muscles), (3) links exist between the task and activation space features, which were revealed by varying the throwing distance, and (4) finally both methods of synergy extraction were able to provide consistent and similar results and are therefore legitimate these methods of extraction.

Lastly, since the identified control representation comprises the use of less control signals than actuators and DoF, it would be useful for synthesizing motions with overactuated or muscle-based characters at a reduced computational cost.

## Figures and Tables

**Table 1:** Assessment of *k*-means clustering quality [49].

Subjective interpretation of the average silhouette value	
$\bar{Sil}$	Proposed interpretation
0.71–1.00	A strong structure has been found
0.51–0.70	A reasonable structure has been found
0.25–0.50	The structure is weak and could be artificial, try additional methods on data set
≤0.25	No substantial structure has been found

**Table 2:** Mean intersubject cross-correlation coefficient per throwing distance.

Mean intersubject cross-correlation coefficient per throwing distance			
DoF	corr_2m_	corr_4m_	corr_7m_
${\dot{q}}_{1}$	0.6681 ± 0.2396	0.7493 ± 0.1579	0.6335 ± 0.2048
${\dot{q}}_{2}$	0.9684 ± 0.0259	0.9494 ± 0.0485	0.9403 ± 0.0529
${\dot{q}}_{3}$	0.9723 ± 0.0207	0.9526 ± 0.0316	0.9329 ± 0.0394
${\dot{q}}_{4}$	0.6025 ± 0.2520	0.6077 ± 0.2634	0.6210 ± 0.1922

${\dot{q}}_{1}$	0.7349 ± 0.1012	0.6290 ± 0.1392	0.4830 ± 0.1317
${\dot{q}}_{2}$	0.6878 ± 0.1257	0.6374 ± 0.1671	0.5588 ± 0.1063
${\dot{q}}_{3}$	0.8170 ± 0.1036	0.8502 ± 0.0880	0.7954 ± 0.0840
${\dot{q}}_{4}$	0.7217 ± 0.1309	0.6540 ± 0.2192	0.7277 ± 0.1226

**Table 3:** Mean intersubject and interdistance cross-correlation coefficients. The representative joint space strategy shown in Figure 9 was correlated across throwing distances.

Mean interdistance cross-correlation coefficients	
DoF	corr_2m,4m,7m_
*q*_1_	0.9171 ± 0.0213
*q*_2_	0.9989 ± 0.0005
*q*_3_	0.9991 ± 0.0004
*q*_4_	0.9315 ± 0.0467

**Table 4:** Synergy coefficients mean intersubject cross-correlation coefficient per throwing distance.

Mean intersubject cross-correlation coefficient per throwing distance			
Synergy coeff.	corr_2m_	corr_4m_	corr_7m_
*c*_1_	0.9129 ± 0.0534	0.9390 ± 0.0320	0.9264 ± 0.0382
*c*_2_	0.8761 ± 0.0727	0.8615 ± 0.0769	0.8702 ± 0.0650

**Table 5:** Synergy coefficients mean interdistance cross-correlation coefficients.

Mean interdistance cross-correlation coefficients	
Synergy coeff.	corr_2m,4m,7m_
*c*_1_	0.9895 ± 0.0035
*c*_2_	0.9833 ± 0.0089
